# Efficacy analysis of mechanical thrombectomy combined with prolonged mild hypothermia in the treatment of acute middle cerebral artery occlusion: a single-center retrospective cohort study

**DOI:** 10.3389/fneur.2024.1406293

**Published:** 2024-07-09

**Authors:** Anqi Wang, Xuan Meng, Qin Chen, YanFei Chu, Qiang Zhou, DongYi Jiang, Zhimin Wang

**Affiliations:** ^1^Department of Neurosurgery, The First Affiliated Hospital of Soochow University, Suzhou, China; ^2^Department of Neurosurgery, Suzhou Kowloon Hospital, Shanghai Jiaotong University School of Medicine, Suzhou, China; ^3^Department of Neurosurgery, Suzhou BOE Hospital, Suzhou, China

**Keywords:** mild hypothermia, occlusion, ischemia/reperfusion, neurological function, mechanical thrombectomy

## Abstract

**Objective:**

To determine the efficacy of mechanical thrombectomy combined with prolonged mild hypothermia compared with conventional treatment in managing acute middle cerebral artery occlusion, and to explore whether extending the duration of hypothermia can improve neurological function.

**Method:**

From 2018 to June 2023, a retrospective analysis was conducted on 45 patients with acute middle cerebral artery occlusion treated at the NICU of Suzhou Kowloon Hospital, affiliated with Shanghai Jiao Tong University School of Medicine. After thrombectomy, patients were admitted to the neurological intensive care unit (NICU) for targeted temperature management. Patients were divided into two groups: the mild hypothermia group (34.5–35.9°C) receiving 5–7 days of treatment, and the normothermia group (control group) whose body temperature was kept between 36 and 37.5°C using pharmacological and physical cooling methods. Baseline characteristics and temperature changes were compared between the two groups of patients. The primary outcome was the modified Rankin Scale (mRS) score at 3 month after surgery, and the secondary outcomes were related complications and mortality rate. Prognostic risk factors were investigated using both univariate and multivariate logistic regression analyses.

**Results:**

Among 45 patients, 21 underwent prolonged mild hypothermia, and 24 received normothermia, with no significant differences in baseline characteristics between the two groups. The duration of mild hypothermia ranged from 5 to 7 days. The incidence of chills (33.3% vs. 8.3%, *p* = 0.031) and constipation (57.1% vs. 20.8%, *p* = 0.028) was significantly higher in the mild hypothermia group compared with the control group. There was no significant difference in mortality rates between the mild hypothermia and the control group (4.76% vs. 8.33%, *p* = 1.000, OR = 1.75, 95% CI, 0.171–17.949). At 3 month, there was no significant difference in the modified mRS (0–3) score between the mild hypothermia and control groups (52.4% vs. 25%, *p* = 0.114, OR = 0.477, 95% CI, 0.214–1.066). Infarct core volume was an independent risk factor for adverse neurological outcomes.

**Conclusion:**

Prolonged mild hypothermia following mechanical thrombectomy had no severe complications and shows a trend to improve the prognosis of neurological function. The Infarct core volume on CTP was an independent risk factor for predicting neurological function.

## Introduction

Interventional thrombectomy has been proven effective for patients with acute ischemic stroke due to major vessel occlusion (1). However, vessel recanalization does not necessarily provide a favorable outcome (2, 3). Due to prolonged time windows and cerebral ischemia/reperfusion injury, approximately 64% of patients with stroke cannot live independently after mechanical thrombectomy (4). Continuous brain damage is still evident in MRI diffusion imaging within 24 h after recanalization (5). Therefore, it is crucial to protect the brain after recanalization, and hypothermia is one of the important strategies (6, 7). In the 1940s, hypothermia was proven beneficial for neurological prognosis following whole-brain ischemia after cardiac arrest and post-traumatic brain injury (8, 9). Similarly, clinical trials on patients with ischemic stroke suggest that hypothermia can alleviate brain edema in the infarct area, reduce intracranial pressure, and lower the mortality rate (10–14). It can also rescue the ischemic penumbra, limiting the growth of the core infarct volume (15). However, the optimal parameters of hypothermia in patients with ischemic stroke, including treatment temperature and duration, remain controversial.

Regarding the temperature, mild hypothermia and moderate hypothermia exhibited the same effects in rat models. Mild hypothermia was superior to moderate hypothermia in terms of side effects and induction (16, 17), mild to moderate hypothermia (32–35°C) can provide protection with much fewer side effects (18). A clinical trial by Hermann et al. (19) which used hypothermia (33.0 ± 1.0°C) combined with decompressive craniectomy, was terminated due to severe adverse reactions, suggesting that moderate hypothermia is detrimental for patients with acute middle cerebral artery stroke. In some clinical trials, mild hypothermia improved neurological prognosis without severe adverse reactions and complications (20, 21). A meta-analysis of numerous studies on post-stroke hypothermia indicated that moderate hypothermia leads to more adverse reactions compared with mild hypothermia (22). It seems that mild hypothermia can achieve the desired effects and is safer. Existing evidence suggests that for every 1°C decrease in body temperature, the side effects increase correspondingly (23).

Animal models of stroke have demonstrated that the protective effects of hypothermia on the brain depend on the timing of initiation and duration of hypothermia (24–26). Moreover, hypothermia needs to be initiated within 1 h to be effective (27), but it is difficult to achieve in most patients with acute stroke. Some scholars believe that the optimal duration of hypothermia for hypoxic–ischemic injury depends on the severity of the injury and when hypothermia is applied. Within a certain range, delays in inducing hypothermia can be compensated by longer durations of hypothermia to achieve the same neuroprotective effects (23, 28, 29), but more studies are needed in this regard. Clinically, the optimal duration of hypothermia is yet unclear, but in patients with global cerebral ischemia and traumatic brain injury (TBI), a longer duration of hypothermia is associated with better outcomes (29–31).

We aimed to explore whether extending the duration of hypothermia can compensate for the damage caused by treatment delays. From 2018 to June 2023, we used prolonged (5–7 days) mild hypothermia (34.5–35.9°C) for treating patients with acute ischemic stroke after thrombectomy. By comparing the mortality rates and prognosis scores of the two groups, we provide insights into the diagnosis and treatment of the disease.

## Methods

### Patients

After obtaining approval from the Ethics Committee of Suzhou Kowloon Hospital, affiliated with Shanghai Jiao Tong University School of Medicine (HG-2023-021), we retrospectively analyzed data from patients diagnosed with acute ischemic stroke who were treated with mechanical thrombectomy at Suzhou Kowloon Hospital from January 2018 to June 2023 (*N* = 178). The inclusion criteria were as follows: (1) aged between 30 and 80 years; (2) presenting with signs of acute stroke upon admission and confirmed unilateral middle cerebral artery occlusion on computed tomography angiography (CTA); (3) National Institutes of Health Stroke Scale (NIHSS) score of ≥10, with the level of consciousness at 1a ≥1; (4) ischemic area >30 mL on computed tomography perfusion (CTP). Exclusion criteria were as follows: (1) undergoing decompressive craniectomy; (2) secondary hemorrhage affecting more than one-third of the infarct area with mass effect; (3) rapid improvement of symptoms; (4) hypothermia not initiated or lasted less than 5 days; (5) unilateral or bilateral pupil dilation during the course of the disease; (6) history of intracranial surgery; (7) severe coagulation disorders or cardiac/pulmonary diseases, rendering the patient unable to tolerate hypothermia. Among patients meeting the inclusion criteria, those who underwent prolonged mild hypothermia were classified as the mild hypothermia group (*N* = 21), and those who received normothermia treatment during the same period were classified as the control group (*N* = 24).

### Mechanical thrombectomy

Mechanical thrombectomy was performed by experienced neurointerventional physicians. Intracranial mechanical thrombectomy (IMT) was conducted using direct aspiration or stent retriever techniques. For cases where reperfusion was not achieved post-thrombectomy, other options, like balloon dilation or stent placement, were available to ensure effective blood flow restoration (modified thrombolysis in cerebral infarction score, mTICI score of 2b/3).

### Mild hypothermia treatment

All patients underwent continuous bladder temperature monitoring using a dual-lumen 16Fr 15 mL catheter (Well Lead Medical Co., Ltd., Guangzhou). After mechanical thrombectomy, all patients were treated by neurocritical care physicians in the neurological intensive care unit (NICU). The patients underwent mechanical ventilation in a sedated and analgesic state, continuously monitored for electrocardiography, and underwent post-pyloric feeding via a nasoduodenal tube, subclavian deep vein catheterization, and continuous but invasive arterial pressure monitoring. A micro-infusion pump continuously released midazolam and remifentanil for ongoing sedation and analgesia while preserving patients’ spontaneous breathing and coughing ability.

In the mild hypothermia group, before induction, a hibernation mixture (chlorpromazine 200 mg, promethazine 200 mg, and pethidine hydrochloride 150 mg, combined with 39 mL of saline to reach a total volume of 50 mL) was continuously infused using a micro-infusion pump. A wrap-around cooling blanket was used for the mild hypothermia group (Changchun ANTAI Medical Instruments Co., Ltd. ZLJ-2000I) to rapidly reach the target temperature. The mild hypothermia treatment was maintained for at least 5 days. Rewarming was conducted as slowly as possible, at a rate of 0.10–0.25°C/h, setting the wrap-around cooling blanket temperature to increase by 1°C every 8 h. After rewarming, the dose of sedatives, analgesics, and hibernation mixture was reduced. The hibernation mixture was discontinued once patients’ body temperature returned to normal. Arterial blood gas analysis was performed every 4 h during the rewarming phase. In the control group, medications and ordinary cooling blankets were used to maintain the core temperature at 36–37.5°C. If the temperature was not controlled using this method, a cooling blanket was used to cool down to normal temperature.

### Conventional treatment

Postoperatively, tirofiban was administered at a continuous infusion rate of 0.1 μg/kg/min for 24 h. At 20 h postoperative, oral administration of aspirin 0.1 g and clopidogrel 75 mg qd was initiated. Each patient was injected with the same dose of edaravone dexborneol and butylphthalide for 2 weeks. Atorvastatin 40 mg qd was administered through the nasogastric tube postoperatively. Mannitol, concentrated sodium, human serum albumin, and other medications were administered based on the patients’ condition. During the treatment period, arterial blood gas analysis was conducted q12h, and complete blood count, coagulation profile, electrolytes, and biochemistry panel were measured at least once every 2 days. All patients underwent daily cranial CT scan in the first 3 days, followed by cranial CT every 3 days after 72 h. Chest CT was conducted every 3–5 days to assess pulmonary infection. Routine bedside transthoracic echocardiography was performed. Fingertip blood glucose was measured q4h, and continuous insulin infusion was administered to maintain blood glucose within the normal range. Enteral nutrition suspension was administered uniformly via a nasogastric feeding pump. A lower limb deep vein ultrasound was conducted weekly to ensure the absence of deep vein thrombosis. Pulmonary multifrequency vibration and lower limb pneumatic compression were used to prevent thrombus formation.

### Data collection, statistics, and outcomes

Baseline data included age, gender, underlying diseases [hypertension, diabetes, atrial fibrillation (AF) Coronary artery disease, and smoking history], delays in initiating mild hypothermia, NIHSS score, Glasgow Coma Scale (GCS) score, core infarct volume, and ischemic penumbra volume on CTP. The primary outcome measure was the modified Rankin Scale (mRS) score at 3 months post-treatment, obtained through telephone follow-ups or hospital medical records. Secondary outcomes included complications, such as chills, hypotension, pulmonary infection, hypokalemia, arrhythmias, deep vein thrombosis, constipation, and hemorrhagic transformation. Hypotension was defined as transient or sustained use of vasopressors. The diagnosis of pulmonary infection was made based on changes in chest CT or positive microbiological culture and concurrent increase in inflammatory markers. Arrhythmias included bradycardia, tachycardia, and AF with rapid ventricular response. AF must have necessitated pharmacological intervention. Constipation was defined as the absence of bowel movement for 3 days or more or the need for prokinetic or laxative medications. These data were obtained from medical records.

Statistical analyses were performed using SPSS 26. Independence between groups was ensured for data analysis, and normal distribution tests were conducted before comparisons. Quantitative data conforming to normal distribution were analyzed using an independent sample *t*-test. Non-normal data were analyzed using non-parametric tests. For qualitative data with *n* > 40, a corrected chi-square test was used when at least one expected value (*T*) was between 1 and 5, and Pearson’s chi-square test was used when all *T-*values were greater than 5. Data following a normal distribution are presented as mean ± SD, while non-normal data are presented as interquartile range (IQR). Univariate analysis and multivariate logistic regression analysis were used to determine the factors affecting the poor neurological outcome. Clinical factors with *p* < 0.05 in univariate analysis were included in multivariate logistic regression analysis. All tests were two-tailed, and a *p*-value <0.05 was considered statistically significant.

## Results

### Patients’ characteristics

**Figure 1:**
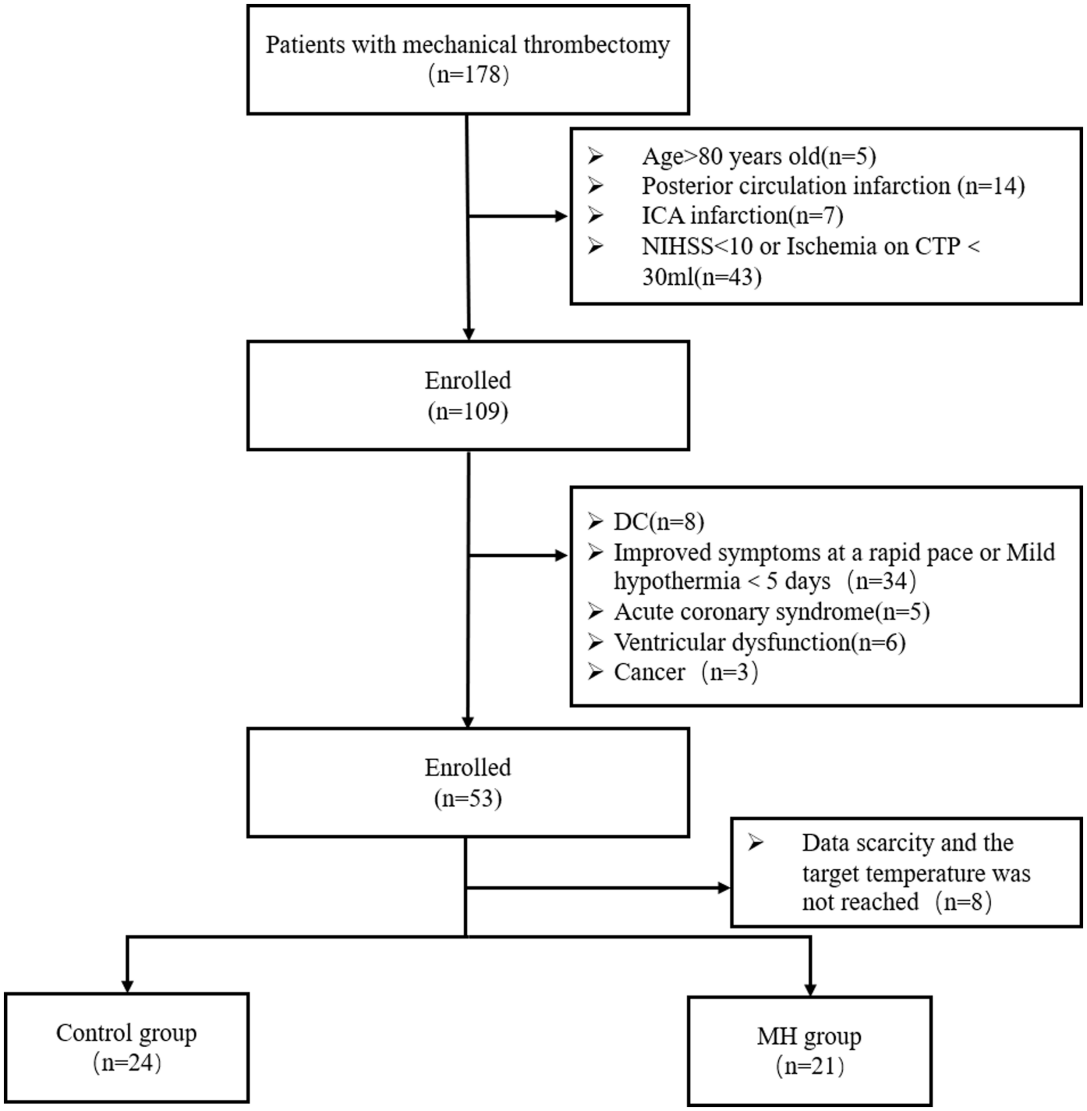
Flow chart of patient profile.

Between 2018 and 2023, 178 patients with acute stroke underwent mechanical thrombectomy at the Shanghai Jiao Tong University School of Medicine. Based on inclusion and exclusion criteria, 53 patients who met the study’s requirements were included. Among them, eight patients were excluded due to the lack of CTP data, lost follow-up data, or not maintaining the target temperature during the mild hypothermia period. The normothermia treatment group (*n* = 24) was considered the control group, and the mild hypothermia group (*n* = 21) was considered the treatment group (Figure 1). The age of patients was similar between the groups (Table 1). Males (32/46, 69.6%) were more prevalent than females. AF was more common in the control group [11/24 (45.8%) cases] compared with the mild hypothermia group [6/21 (28.6%) cases]. There were no significant differences in the baseline characteristics of patients (Table 1).

**Table 1:** Baseline characteristic of the patients.

Characteristic	HM group (*n* = 21)	Control group (*n* = 24)	*p-*value
Age, y, mean ± *SD*	60.1 ± 10.7	61.4 ± 12.5	0.933
Men, *n* (%)	14(66.7)	18(75)	0.775
Infarct volume on CTP, ml, median (IQR)			
Infarct core	14.3(11.9–21.4)	22.4(13.3–28.9)	0.065
Ischemic penumbra	78.5(68.4–92.8)	75.7(59.7–83.8)	0.191
NIHSS, median (IQR)	15(10.5–18)	14(11.3–17.8)	0.863
GCS, median (IQR)	11(8.5–12.5)	10(9–12)	0.640
Premorbid mRS, median (IQR)	0(0–0)	0(0–0)	0.181
Infarct related artery, *n* (%)			0.526
*M*_1_	13(61.9)	17(38.1)	
*M*_2_	8(70.8)	7(29.2)	
Underlying diseases, *n* (%)			
Hypertension	15(71.4)	14(58.3)	0.360
Diabetes mellitus	3(14.3)	6(25)	0.601
Atrial fibrillation	6(28.6)	11(45.8)	0.233
Coronary artery disease	2(9.5)	6(25)	0.355
Smoking	11(52.4)	11(45.8)	0.661
Onset to IMT time, h, median (IQR)	5(4–6.2)	6(4.5–7.8)	0.390

### Temperature management

**Figure 2:**
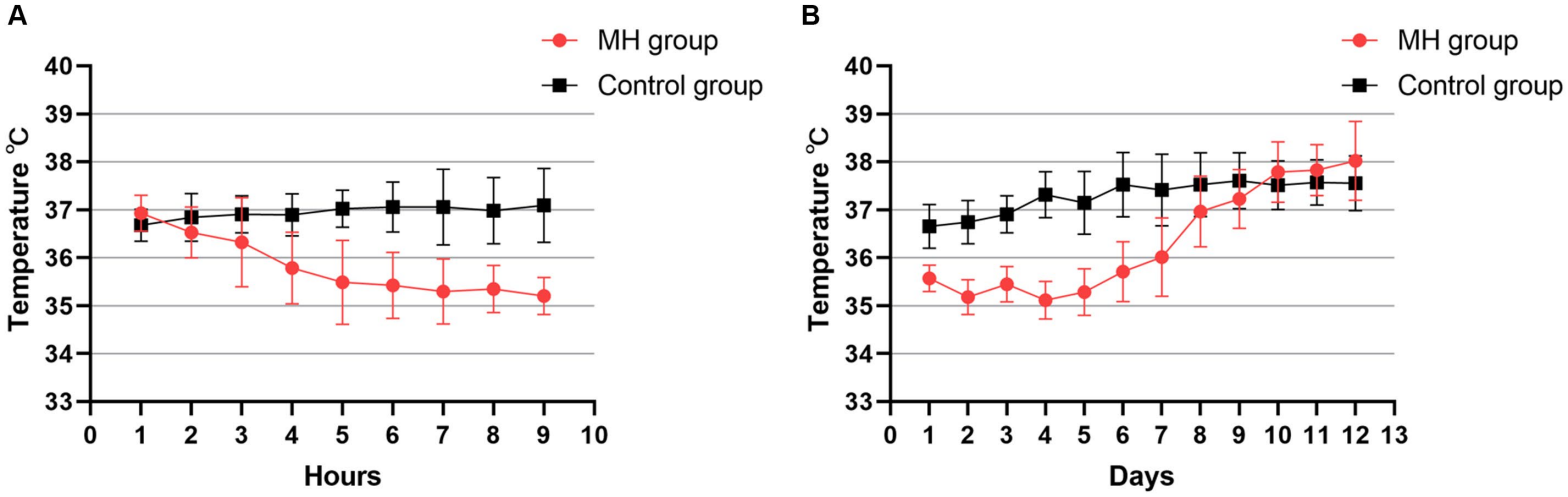
**(A)** Line plot of temperature changes in the two groups during the first 8 h of the first day of hypothermia initiation. Temperature was the bladder temperature recorded hourly by the patient. **(B)** Temperature changes were recorded continuously for 12 days after starting hypothermia. Each point in the graph consists of the daily mean temperature and the value of the standard deviation for all patients in the two groups.

Figure 2A presents body temperature changes in both patient groups 8 h after hospital admission. In the mild hypothermia group, patients reached the target temperature within 2–6 h of initiating hypothermia. There was no significant difference in the baseline temperature between the groups (36.9 ± 0.4°C in the mild hypothermia group vs. 36.7 ± 0.3°C in the control group, *p* = 0.07). Figure 2B shows the temperature change curves for both groups during 12 days of hospital stay. This graph reveals that the duration of hypothermia in the mild hypothermia group was maintained for 5–7 days.

### Complications

The incidence of chills, hypokalemia, constipation, and pneumonia was higher in the mild hypothermia group compared with the control group (Table 2). There was a significant difference in the incidence of chills in the mild hypothermia group compared with the control group (33.3% vs. 8.3%, *p* = 0.031). No patients in the mild hypothermia group needed the termination of treatment due to uncontrollable chills. Chills were well-controlled with enhanced sedation and muscle relaxants. The incidence of constipation in the mild hypothermia group was significantly higher than in the control group (57.1% vs. 27.8%, *p* = 0.028). The incidence of hypokalemia was 52.4% (11/21) in the mild hypothermia group and 29.2% (7/24) in the control group. Hypokalemia was corrected to normal ranges either through nasogastric or intravenous supplementation. There were no cases with severe hypokalemia whose hypokalemia was difficult to correct or necessitated the termination of hypothermia. All seven instances of hemorrhagic transformation were minor bleedings without significant mass effect. After detecting bleeding on CT scan, aspirin and clopidogrel were immediately discontinued, and neutral treatment was adopted. The rate of pneumonia in the mild hypothermia group was higher than that in the control group (71.4% vs. 54.2%), but this difference was not statistically significant (*p* = 0.233). Due to the inclusion of AF episodes in the arrhythmia category and the higher number of patients with AF in the control group, the incidence of arrhythmia was higher in the control group compared with the mild hypothermia group (38.1% vs. 54.2%), yet without statistical significance (*p* = 0.281).

**Table 2:** Complications.

Complications	HM group (*n* = 21)	Control group (*n* = 24)	*P-*value
Chills, *n* (%)	7(33.3)	1(8.3)	0.031
Hypotension, *n* (%)	4(19.0)	4(16.7)	1.000
Pneumonia, *n* (%)	15(71.4)	13(54.2)	0.233
Hypokalemia, *n* (%)	11(52.4)	7(29.2)	0.113
Arrhythmias, *n* (%)	8(38.1)	13(54.2)	0.281
DVT, *n* (%)	1(4.8)	2(8.3)	1.000
Constipation, *n* (%)	12(57.1)	5(20.8)	0.028
Hemorrhagic transformation, *n* (%)	2(9.5)	5(20.8)	0.335

### Mortality rates

At 3 month, the mortality rate was 8.33% (2/24) in the control group. Two male patients aged 79 and 78 years died in this group. Due to recurrent pulmonary infections, these patients re-entered the NICU for mechanical ventilation and ultimately chose to discontinue treatment and were discharged due to severe infection and respiratory failure. In the mild hypothermia group, the mortality rate was 4.76% (1/21). A 72-year-old female patient died in this group. The cause of death was similar to the aforementioned cases.

### Outcome

**Figure 3:**
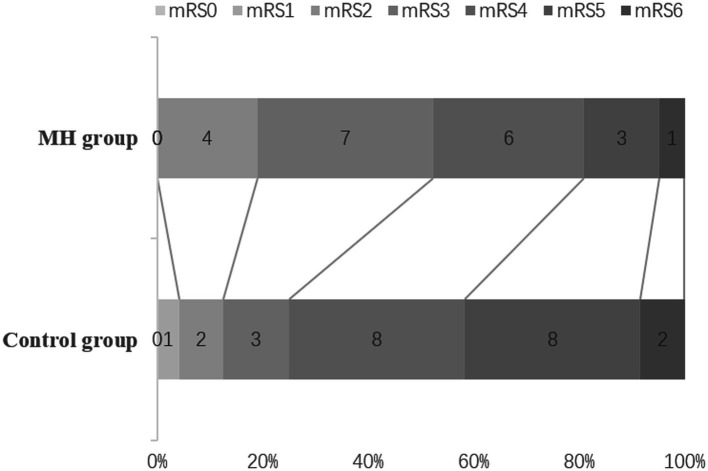
mRS at 3 months. The rates of mRS 0–3 in the MH, and control groups were 52.4% (13/21), 25% (6/24), respectively, but there was no statistical difference (*P* = 0.114; OR = 0.477, 95%Cl, 0.214 ~ 1.066). The mortality rates in the MH and Control groups were 4.76% (1/21), 8.33% (2/24), respectively.

The mRS scores were evaluated 3 month, the proportion of mRS0-3 was counted. The number of patients with mRS0-3 was 11/21 (52.4%) in the mild hypothermia group and 6/24 (25%) in the control group (Figure 3). The proportion of mRS0-3 was higher in the mild hypothermia group compared with the control group, but the difference was not statistically significant (*p* = 0.114; OR = 0.477, 95% CI, 0.214–1.066).

### Multivariate logistic regression analysis for poor prognosis

**Figure 4:**
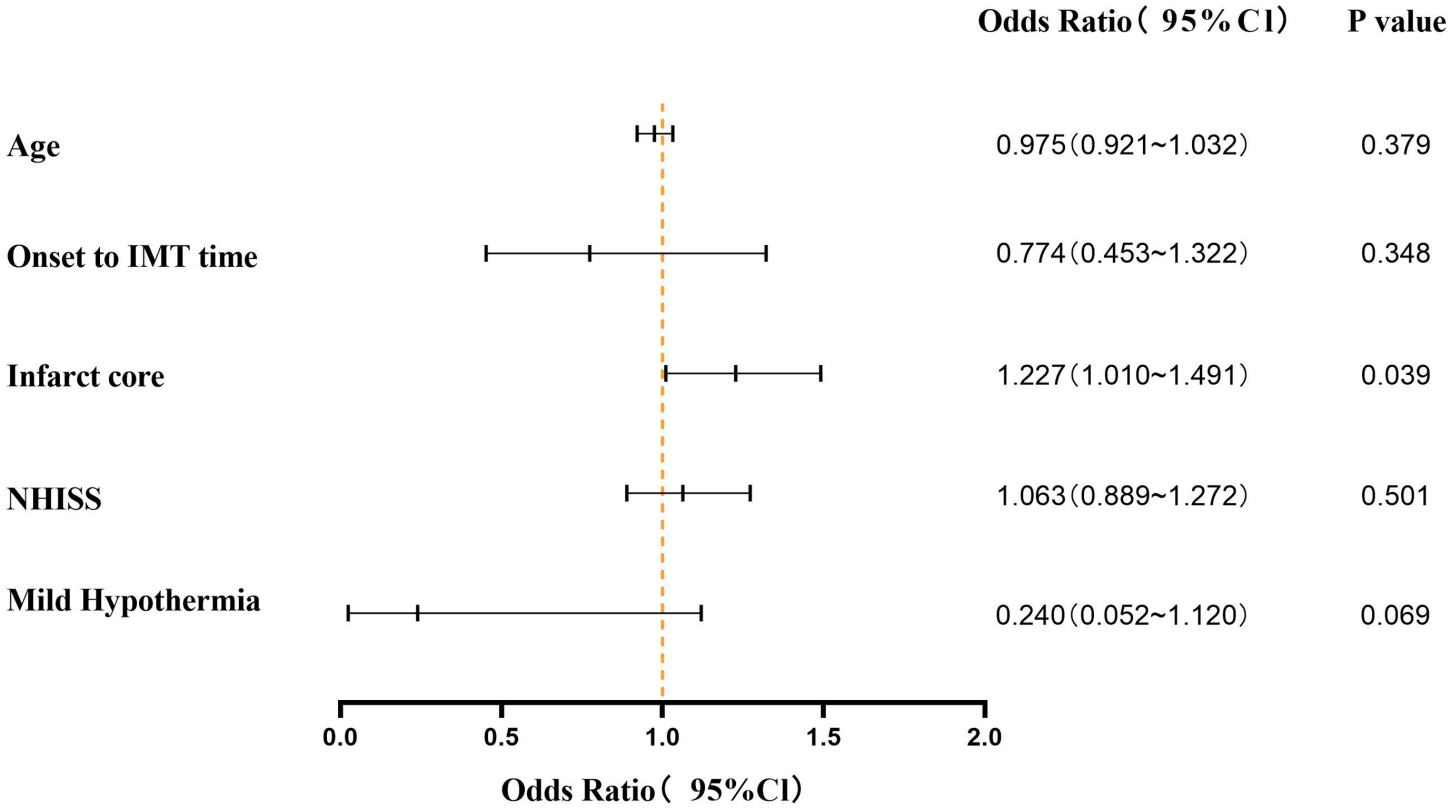
Multivariate logistic regression analysis for poor outcome (mRS>3).

Univariate logistic regression analysis showed that age, onset to IMT time, infarct core, NIHSS and hypothermia therapy were statistically significant (*p* < 0.05). Multivariate logistic regression analysis showed that the infarct core was an independent factor affecting the poor mRS Score (*p* = 0.039; OR = 1.227, 95%Cl, 1.010–1.491) (Figure 4).

## Discussion

Hypothermia, in most clinical trials, as a treatment method for intracranial reperfusion injury has not demonstrated significant improvement in the prognosis of neurological function. However, there is a delay in initiating hypothermia treatment. Whether extending the duration of hypothermia can improve neurological function was the primary aim of our retrospective analysis. Combining numerous reports on hypothermia and our previous experience, the target temperature for hypothermia treatment was shifted from deep or moderate hypothermia to mild hypothermia. Mild hypothermia not only retains the therapeutic effect but also reduces adverse reactions. Additionally, most patients with acute stroke are elderly and may not tolerate moderate hypothermia. As mild hypothermia may not effectively reduce intracranial pressure, we excluded patients needing decompressive craniectomy for extensive infarct cerebral edema. We aimed to protect neurons and improve neurological function, not primarily to reduce intracranial pressure. Hence, we chose mild hypothermia (34.5–36°C) to explore its neuroprotective effect. The patients included in our study all had varying degrees of consciousness impairment and could not undergo interventional thrombectomy with local anesthesia, thus necessitating general anesthesia. This helped family members to accept prolonged hypothermia when the patient returned to the NICU after the surgery. Although there have been instances of hypothermia treatment in conscious states (32, 33), we have not yet attempted prolonged hypothermia.

Prolonged mild hypothermia treatment at 34.5–35.9°C was safe in our study, with no severe complications. The incidence of chills was significantly higher in the mild hypothermia group compared with the control group (33.3% vs. 8.3%, *p* = 0.031). However, this was controlled in the short term by increasing the dosage of the hibernation mixture, deepening sedation and analgesia, and using muscle relaxants. Surface cooling for mild hypothermia treatment inherently causes physiological chills, but timely alleviation or elimination of chills is the key to successful hypothermia. There was a significant statistical difference in the incidence of constipation between the groups (57.1% vs. 20.8%, *p* = 0.028) (Table 2). The higher incidence of constipation in the mild hypothermia group could be due to the use of higher doses of anesthetic drugs, leading to reduced gastrointestinal motility and constipation. There were no cases of severe abdominal distension under hypothermia treatment. The incidence of pneumonia (71.4% vs. 54.2%) and hypokalemia (52.4% vs. 29.2%) were higher in the mild hypothermia group than in the control group but without statistical significance. Among patients with pneumonia in the mild hypothermia group, seven experienced varying degrees of atelectasis, which might be associated with prolonged sedation, analgesia, and hibernation state during hypothermia. Additionally, intravenous sedatives or muscle relaxants might have been necessary to promptly alleviate chills, thereby increasing the depth of anesthesia. Excluding the three deceased patients, pulmonary infections were generally controllable and did not lead to unmanageable infections. In two previous clinical trials investigating short-term hypothermia therapy (34, 35), ICTuS-L used an endovascular cooling technique after intravenous thrombolysis, and COOLAID has surface cooling and intravascular cooling in two ways. While both of these clinical trials targeted a hypothermic state of 33°C and lasted for 24 h, it is noteworthy that the systemic nature of cooling led to an inevitably high incidence of pneumonia [ICTuS-L (50% vs. 10%) and COOLAID (35% vs. 9%)]. While the incidence of pneumonia was higher in prolonged mild hypothermia in our study compared with these two short-term hypothermia. Although it has been reported that the incidence of pneumonia in intensive care units is approximately 70% even under normal temperature conditions (36), temperature changes in the two groups reveals that patients which in mild hypothermia group may experience more severe inflammatory responses compared to the control group after rewarming, the baseline body temperature of patients in the hypothermia group after rewarming was approximately 1°C higher than that of the control group (Figure 2B). Through a retrospective analysis of cases, we found that the elevated incidence of pneumonia in the mild hypothermia group led to fever, and in some cases, even hyperthermia. The immunosuppression under hypothermic conditions, along with prolonged sedation, analgesia, and intubation with mechanical ventilation, may contribute significantly to the high prevalence of pulmonary infections. It is worth further exploring whether modulating the immune system or administering antibiotics earlier during long-term mild hypothermia therapy could reduce the incidence of pneumonia.

All three deceased patients succumbed to recurrent pulmonary infections. They were readmitted to NICU for mechanical ventilation and ultimately died due to respiratory failure and exacerbated infection. The ages of these patients ranged from 72 to 79 years, and they had poor baseline conditions. Two males aged 79 and 78 died in the control group, and a 72-year-old female died in the mild hypothermia group. In the post-acute phase, the inability of patients to care for themselves and prolonged bed rest often make pneumonia a major factor in increasing their mortality. Whether prolonged mild hypothermia is unsuitable for elderly patients requires further subgroup analyses and larger sample sizes. Eight patients (38.1%) in the mild hypothermia group and 13 patients (54.2%) in the control group had a history of AF (Table 1). Since AF was also considered an arrhythmias, the control group had a higher proportion of arrhythmias. Hypotension occurred more frequently in the mild hypothermia group (19%) compared with the control group (16.7%). In the mild hypothermia group, the primary causes of hypotension were sedatives, analgesics, and hibernation mixture during the hypothermia induction phase, with no instances of hypotension during the rewarming phase. In the control group, some patients experienced hemodynamic instability during AF episodes, leading to hypotension. Hypothermia can affect coagulation and platelet function, but coagulation and platelet dysfunction generally occur at temperatures below 33°C (37–39). Due to the use of heparin during the surgery, postoperative use of tirofiban, and bridging with antiplatelet medications, this study did not include coagulation function and thromboelastography.

Although surface cooling is relatively easy to implement, it is systemic cooling, which inevitably brings more side effects. In our study, even mild hypothermia (34.5–35.9) were associated with significantly higher rates of chills, decreased gastrointestinal function, and a relatively higher incidence of pneumonia, which will prolong the treatment time and Cost of hospitalization, it can be fatal in elderly patients. Endovascular cooling techniques would be more accurate and tolerable but less widely available. Selective cooling of the head surface will avoid systemic complications, but in patients with an intact skull, the target temperature cannot be achieved.

Although several clinical studies suggested that mild hypothermia may improve neurological outcomes and reduce infarct volume, only three clinical studies indicated that mild hypothermia can significantly improve the neurological prognosis of patients with acute cerebral infarction (21, 40, 41). In our study, mRS score of 0–3 ratio was higher in the mild hypothermia group than in the control group (52.4% vs. 25%, *p* = 0.114), which is consistent with the results of most clinical trials. Due to a higher proportion of good prognosis compared with the control group, we believe that prolonged hypothermia may improve neurological outcome. Hypothermia treatment is not a single therapeutic modality but a comprehensive intervention, including prolonged sedation and analgesia, mechanical ventilation, meticulous airway management, and extensive monitoring of various indicators, requiring an expert medical and nursing team.

### Limitations

Our study was a single-center retrospective cohort study. Due to the low incidence rate of the disease, it was challenging to collect a large sample size, making it difficult to draw definitive conclusions. One of the indicators of mild hypothermia therapy is brain temperature. Bladder temperature does not represent intracranial temperature. Especially when the cooling method is surface cooling. Using an intracranial pressure (ICP) monitor allows more accurate monitoring of brain temperature, ICP, and cerebral perfusion pressure (CPP) values. However, this requires consent. Additionally, this was a retrospective analysis, not a prospective randomized controlled study. Neurointensivists in the selection of patients for the mild hypothermia group may have introduced a selection bias, potentially affecting the results.

## Conclusion

This study indicates that prolonged mild hypothermia following mechanical thrombectomy had no severe complications and shows a trend to improve the prognosis of neurological function. Larger infarct core volume predicted worse neurologic outcome. These findings must be validated in multicenter randomized controlled trials (RCTs).

## Data availability statement

The raw data supporting the conclusions of this article will be made available by the authors, without undue reservation.

## Ethics statement

The studies involving humans were approved by the Ethics Committee of Suzhou Kowloon Hospital. The studies were conducted in accordance with the local legislation and institutional requirements. Written informed consent from the patients or patients legal guardian/next of kin was not required to participate in this study in accordance with the national legislation and the institutional requirements.

## Author contributions

AW: Data curation, Formal analysis, Investigation, Writing – original draft. XM: Investigation, Writing – review & editing, Formal analysis, Validation. QC: Data curation, Supervision, Writing – review & editing. YC: Conceptualization, Supervision, Writing – review & editing. QZ: Supervision, Investigation, Writing – review & editing. DJ: Conceptualization, Supervision, Investigation, Resources, Writing – review & editing. ZW: Supervision, Funding acquisition, Writing – review & editing.
